# Projections of Primary and Revision Shoulder Arthroplasty until 2040: Facing a Massive Rise in Fracture-Related Procedures

**DOI:** 10.3390/jcm10215123

**Published:** 2021-10-31

**Authors:** Alexander Klug, Eva Herrmann, Sebastian Fischer, Reinhard Hoffmann, Yves Gramlich

**Affiliations:** 1 Abteilung für Unfallchirurgie und Orthopädische Chirurgie, BG Unfallklinik Frankfurt am Main gGmbH, Friedberger Landstrasse 430, 60389 Frankfurt am Main, Germany; sebastian.fischer@bgu-frankfurt.de (S.F.); reinhard.hoffmann@bgu-frankfurt.de (R.H.); yves.gramlich@bgu-frankfurt.de (Y.G.); 2 Institut für Biostatistik und Mathematische Modellierung, Goethe-Universität Frankfurt am Main, Theodor-Stern-Kai 7, 60596 Frankfurt am Main, Germany; herrmann@med.uni-frankfurt.de

**Keywords:** shoulder arthroplasty, reverse shoulder arthroplasty, proximal humerus fracture, hemiarthroplasty, projections, revision

## Abstract

Although the demand for shoulder arthroplasties has reached its highest number worldwide, there remains a lack of epidemiologic data regarding recent and future trends. In this study, data for all shoulder arthroplasties (hemiarthroplasty, reverse/anatomic shoulder arthroplasty) from the nationwide inpatient statistics of Germany (2010–2019) and population forecasts until 2040 were gathered. A Poisson and a negative binomial approach using monotone B-splines were modeled for all types of prostheses to project the annual number and incidence of primary and revision arthroplasty. Additionally, trends in main indicators were also gathered and expected changes were calculated. Overall, the number of primary shoulder replacements is set to increase significantly by 2040, reaching at least 37,000 (95% CI 32,000–44,000) procedures per year. This trend is mainly attributable to an about 10-fold increased use of fracture-related reverse shoulder arthroplasty in patients over 80 years of age, although the number of procedures in younger patients will also rise substantially. In contrast, hemiarthroplasties will significantly decrease. The number of revision procedures is projected to increase subsequently, although the revision burden is forecast to decline. Using these country-specific projection approaches, a massive increase of primary and revision shoulder arthroplasties is expected by 2040, mainly due to a rising number of fracture-related procedures. These growth rates are substantially higher than those from hip or knee arthroplasty. As these trends are similar in most Western countries, this draws attention to the international issue, of: if healthcare systems will be able to allocate human and financial resources adequately, and if future research and fracture-prevention programs may help to temper this rising burden in the upcoming decades.

## 1. Introduction

With the baby boomer generation reaching 65 years of age, the socioeconomic issues of an aging population, with higher incidences of degenerative joint diseases [1] and fractures, will certainly affect inpatient care. While the increase in the number of hip and knee arthroplasties is predicted to slow down over the next decades [2,3,4,5,6,7], only a few studies have examined national trends for upper extremity arthroplasties [8,9]. However, in contrast to hip and knee arthroplasty, the demand and impact of shoulder arthroplasty have been underestimated in the past, probably due to a smaller number of arthroplasties performed per year. In the United States, which is commonly used as a reference in other orthopedic studies, current growth rates for shoulder arthroplasties are reported to be comparable with, or even higher than the growth rates for total hip and knee procedures [8,10]. Furthermore, the projected demand for shoulder arthroplasty is anticipated to increase more rapidly over the next decade based on the future demographic development of the U.S. [9]. However, in contrast to the United States, Germany and many other Western (European) countries face population declines in the near future due to lower birth and immigration rates, which are unable to make up for the aging of the population. Based on current projections, many other countries will likely be heading in the same direction within the next few decades [11,12]. As the incidence of shoulder arthroplasty is higher for older patients, an increased burden in the future has to be expected, as age-related diseases, like osteoarthritis, osteoporosis, frailty-syndrome or injuries are becoming increasingly important. In Germany, the incidence of shoulder arthroplasty is amongst the highest around the world [13], due to a relatively old population and a social healthcare system, which provides almost unlimited access to all parts of orthopedic treatment. However, as the working population is shrinking and increasingly aging, the healthcare system faces the challenge of higher demand and costs.

Furthermore, newer implant designs and expanded indications, such as the use of reverse total shoulder arthroplasty (rTSA) for proximal humerus fractures [14,15], have also led to the rising surgical volume of shoulder arthroplasties in recent years. A shift towards total shoulder arthroplasty (TSA) and away from hemiarthroplasty (HA) procedures have also been reported, presumably because of better outcomes both in degenerative and traumatic conditions [16,17]. However, the contribution of rTSA to future projections of shoulder arthroplasties has not been evaluated yet. Furthermore, the revision burden of these procedures appears to be rising as well [8], which is of particular concern because revision surgery tends to be more complex and cost-intensive than primary arthroplasty [18]. 

Therefore, the aim of this study was to provide a reliable projection of the future need for primary and revision shoulder arthroplasty to assist orthopedic surgeons, politicians, healthcare providers and other stakeholders (insurances, industry) in providing enough human and financial resources to maintain the current standard of care.

## 2. Materials and Methods

### 2.1. Data Collection

An analysis of the data from the national inpatient statistics of Germany (DeStatis) was conducted. This database includes all annual inpatient treatment reports from all German hospitals and medical institutions, making this study a nationwide survey (except military and psychiatric facilities). The data are based on the International Statistical Classification of Diseases and Related Health Problems, Tenth Edition (ICD-10) and the German procedure classification system (OPS), which is the official classification system for encoding surgical procedures in Germany. These statistics contain anonymized data sustaining plausibility checks and data validation on a medical and economic level. All cases reported between 2010 and 2019 were analyzed based on the corresponding OPS codes in its most recent version [19], as well as their associated ICD diagnosis. Data after 2019 was intentionally excluded because of the interference due to the COVID-19 pandemic, which led to a massive reorganization of the medical system in 2020 and early 2021. All patients with shoulder arthroplasty, including either anatomic or reverse arthroplasty and all hemiarthroplasties, as well as all revision shoulder arthroplasties and explanations, were identified. During the study period, no coding changes were performed. Age was categorized in the following groups: <55, 55–59, 60–64, 65–69, 70–74, 75–79, 80–84, 85–89, and older than 90 years.

Population data was available from official population projection statistics until 2040 [20]. These population projections consider the future mortality and increased life expectancy for the oldest population groups, and the immigration rate. 

### 2.2. Data Analysis

Data from 2010 to 2019 (baseline years) and population forecasts up to the year 2040 were used to project the annual incidence of shoulder arthroplasty in Germany. A linear (Poisson, “classic approach”) regression analysis was performed to estimate the expected incidence with the calendar year, sex, and patient age as covariates. The incidence was calculated by dividing the estimated number of arthroplasties for the national total and for each age subgroup by the corresponding official population forecast. An offset variable for the size of the population was chosen to ensure that the procedural number did not exceed the total population number. To overcome overdispersion problems that could result in an underestimation of the variance, we used a robust sandwich covariance matrix estimator for variance calculation. To minimize the error of variance underestimation of the estimated parameter because of overdispersion, we applied a quasi-Poisson regression to our data in accordance with the theory of quasi-likelihood. 

As regressions based on logarithm, or an exponent, like Poisson, will only fit optimally when that is the exact nature of the true relationship, they might not be economically plausible, as in principle the projected counts can rise to infinity. In contrast, it seems quite reasonable to imagine that there might rather be an asymptotic or curvilinear relationship. To overcome this issue, an alternative estimate of the TSA projections was also determined by fitting the incidence rates (counts per 100,000 persons and year) with a negative binomial regression model using a monotone B-spline approach (“new approach”) for modeling time effects and accounting for respective gender and age groups. Splines are used in statistics in order to mathematically reproduce flexible shapes. Knots are placed at several places within the data range, to identify the points where adjacent functional pieces join each other. The advantage of using splines for yearly data compared to the traditional approach is the more accurate curve estimation for the nonlinear trend changes and the simple way of modeling interactions between the time variables. 

To compare the prediction accuracy of each model, the dataset was then split into training (years 2010–2017) and testing subsets (years 2018–2019). Both models were analyzed regarding common forecast accuracy measurement instruments (mean squared error (MSE), root mean squared error (RMSE), the mean absolute percentage error (MAPE) and Theil’s U inequality coefficients, of which the first (U1) is a measure of forecast accuracy and the second (U2) is a measure of forecast quality [21]), which showed lower and thus more accurate values for the negative binominal approach using monotone B-splines (Table 1).

Because of the anonymization of the diagnosis-related group DRG data, arthroplasty patients who underwent a revision (replacement or explanation) could not be individually followed and therefore, actual revision rates could not be calculated. Instead, we estimated the revision burden (RB) by dividing the number of revisions in the form of replacements or explanations by the number of all primary and revision shoulder arthroplasties (HA and TSA), as described previously [22]. Future projections for revision arthroplasty were also calculated, as mentioned above. For better comparison, a “constant rate” approach was used, based on the average revision burden during the baseline years and the projections of shoulder replacements in 2040.

All statistical analyses were performed using R Version 3.4.0 (R Development Core Team, The R Foundation for Statistical Computing, Vienna, Austria).

## 3. Results

### 3.1. Historical Data: Baseline Years 2010–2019

**Figure 1:**
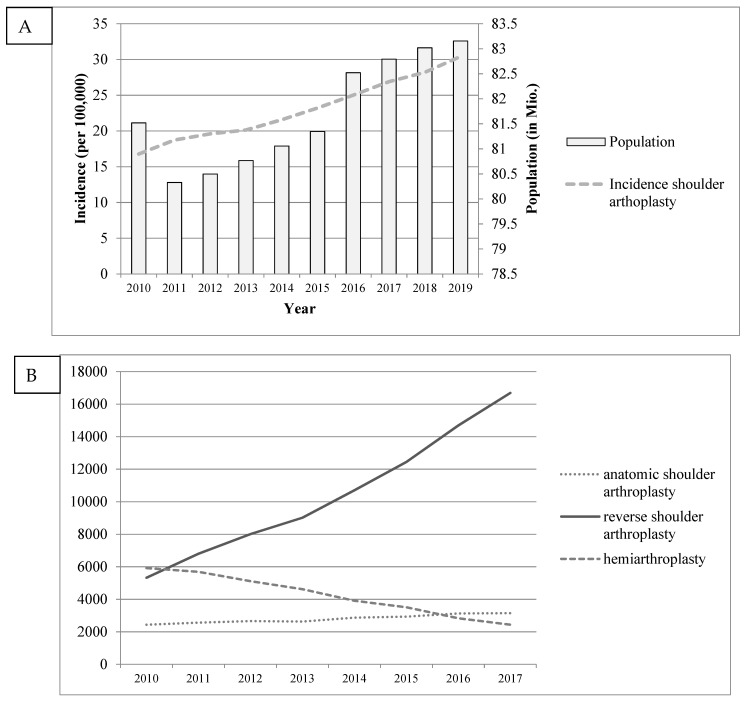
German population, shoulder arthroplasty incidence (**A**) and type of procedure (**B**) from 2010 to 2017.

**Figure 2:**
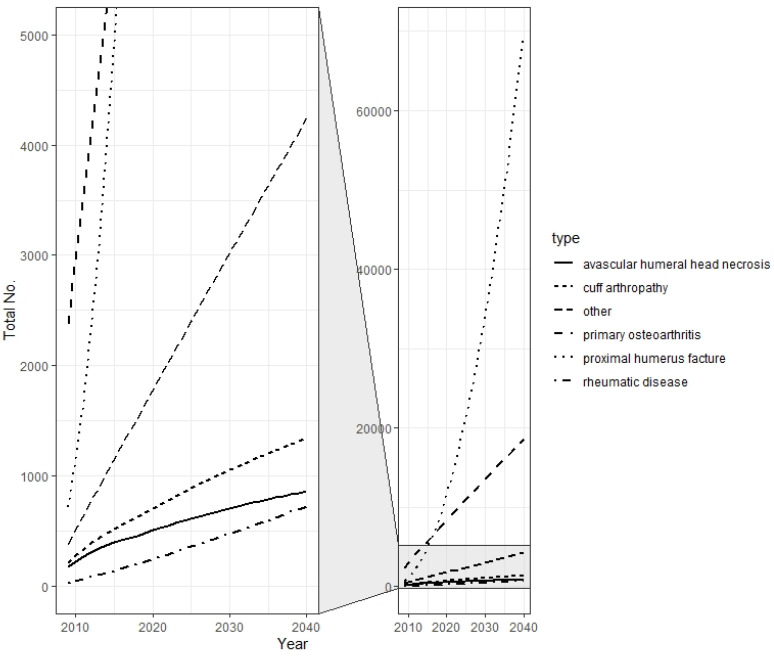
Historical and projected main indications for reverse shoulder arthroplasty from 2010 to 2040.

The procedural volume of primary shoulder arthroplasties increased by approximately 14% each year from 13,678 to 25,294 patients, amounting to an incidence rate of 30.4 per 100,000 in 2019 (Figure 1). This development has mainly been attributed to increased utilization of rTSA, for which the number of procedures has almost quadrupled since 2010. In addition, the use of HA decreased by over 70%, while the number and incidence of anatomic total shoulder arthroplasty procedures (aTSA) showed almost steady progress over time. Regarding the demand in different age groups, in 2010, 5.2% of all primary shoulder arthroplasties and only 1.8% of all rTSA procedures were performed in younger patients (55 years or younger). By the end of 2019, the relative size of this population had decreased to 4.1% and 1.4%, respectively. Overall, an enormous increase in fracture-related arthroplasty could be identified, with the number of fracture-related rTSA showing an almost ten-fold increase during that time span (Figure 2). Additionally, the overall number of osteoarthritis (OA)-related procedures also almost tripled, with rTSA becoming increasingly important compared to aTSA or HA.

During the study period, 19,190 revision procedures (replacements or explanations) were undertaken, which included 5748 revisions for HA and 13,241 for TSA, with an overall RB of 12.9% for HA and 7.0% for TSA. However, we identified a slight RB decrease for TSA, while the RB for HA significantly increased (from 7.1% to 18.4%). For both procedure types, a 1.2-fold higher relative risk for revision was calculated for males compared with females.

### 3.2. Projection of Primary Shoulder Arthroplasty: Years 2020–2040

**Figure 3:**
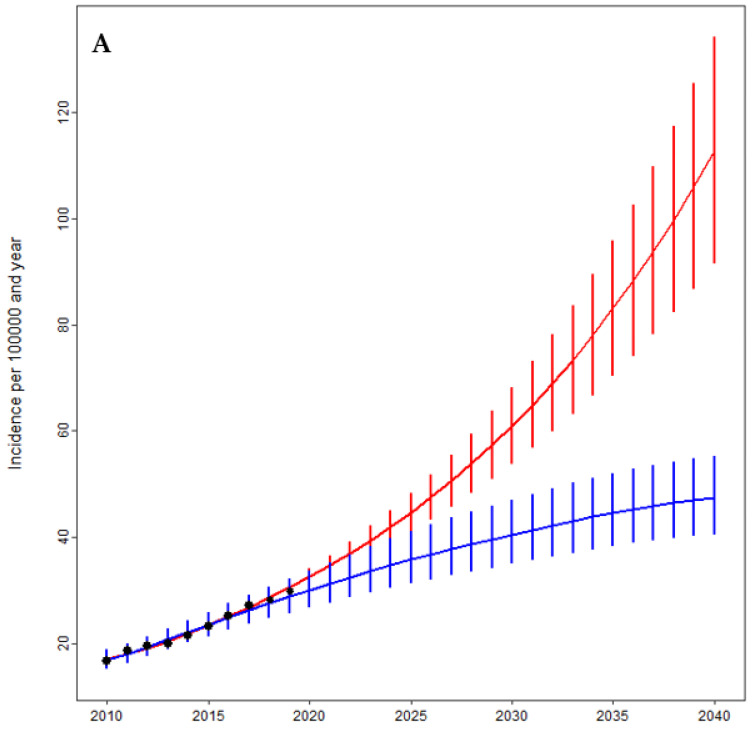

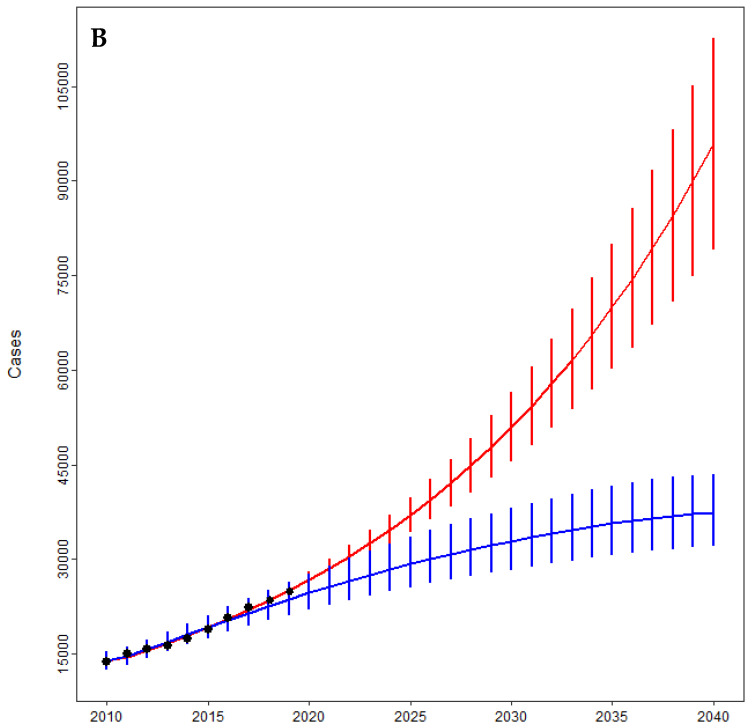
Projections of the incidence (**A**) and total number (**B**) of shoulder arthroplasties by the year 2040 based on a Poisson (red) and a negative binomial regression model using a monotone B-spline approach (blue). The black points represent the historical numbers.

Based on our quasi-Poisson projection model, the annual number of shoulder replacements is estimated to grow to 95 × 10^3^ (95% CI 79–112 × 10^3^) by 2040 (Figure 3). The forecasted incidence rate was projected to be 112 (95% CI 91–134) per 100,000 German residents, resulting in a more than seven-fold growth from 2010 to 2040. Additionally, we used a negative binomial approach with monotone B-splines to achieve a different projective view on the future demand for shoulder arthroplasty. This model projected the annual number of shoulder replacements to rise to 37 × 10^3^ (95% CI 32–44 × 10^3^) by 2040, leading to an incidence rate of 47 (95% CI 41–55) per 100,000 inhabitants, which still represents a rise of approximately 175% since 2010. 

### 3.3. Projections of Shoulder Arthroplasty as a Function of Age

**Figure 4:**
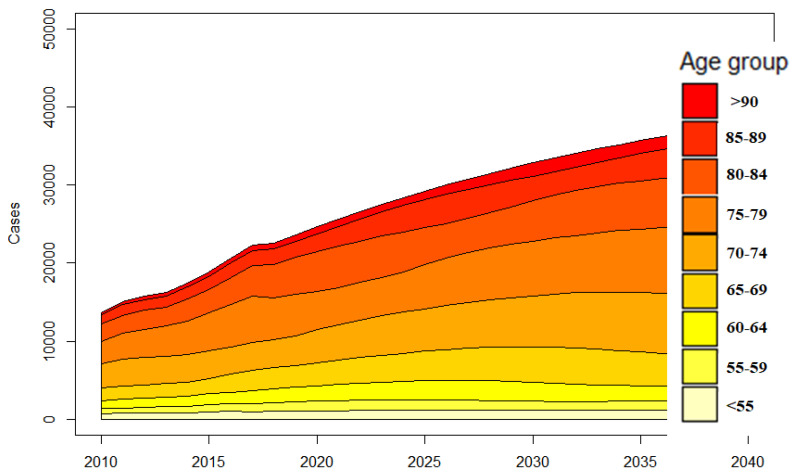
Reported and predicted case numbers per age group from 2010 to 2040.

**Figure 5:**
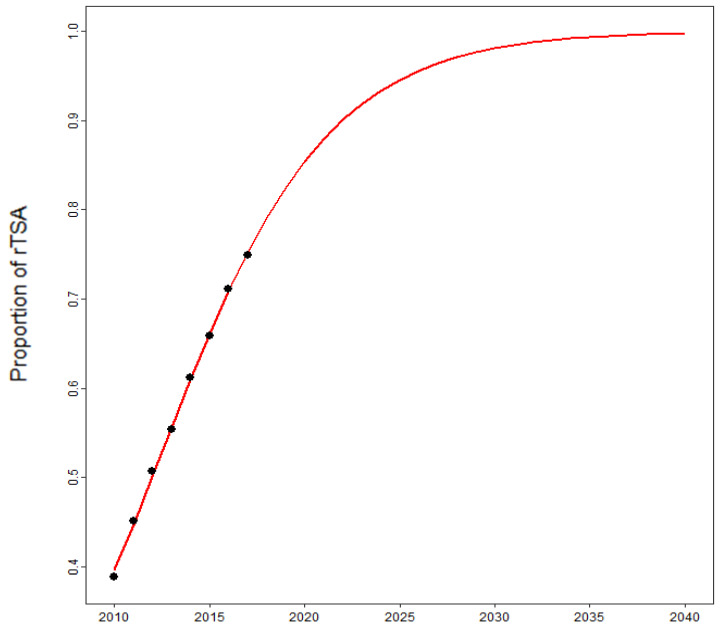
Projected share of reverse total shoulder arthroplasties (rTSA) in total shoulder arthroplasties from 2010 to 2040.

Considering age, the incidence rates almost doubled in each age group, while the total amount of shoulder replacements showed the highest increase in older patients (Figure 4). This increase can mainly be attributed to the rising utilization of rTSA, which will be responsible for over 90% of all shoulder replacements in 2040 if our projections hold true (Figure 5). These arthroplasties will be performed due to a massive increase in fracture-related scenarios, which could reach an eight-fold increase by 2040 (Figure 2). 

Despite a simultaneously rising number of TSA in younger patients, the demand for primary shoulder arthroplasties among patients aged 55 y or younger was projected to decrease from 5.2% in 2010 to 3.2% (95% CI, 2.7–3.6) of all recipients by 2040.

### 3.4. Projections of Revision Shoulder Arthroplasty

**Figure 6:**
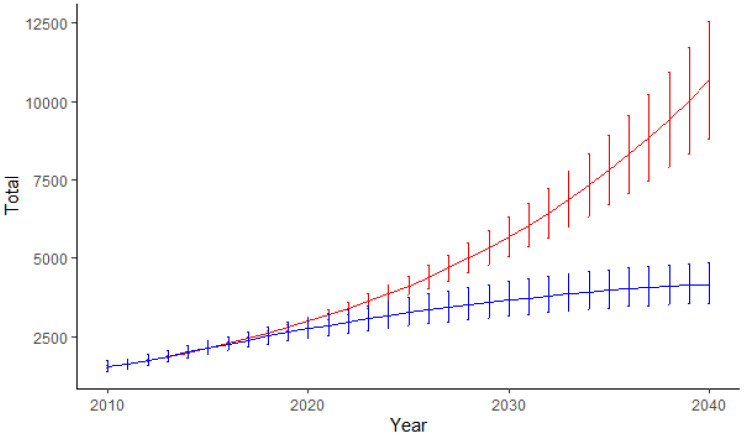
Projections for revision shoulder arthroplasty procedures using a “constant-rate” approach based on a Poisson (red) and a negative binomial regression model using a monotone B-spline approach (blue).

Along with the rising number of primary replacements, the number of revision procedures is projected to increase as well. Based on our models, this number will reach its maximum of approximately 4000 (upper 95% CI limit: 9000) procedures in 2040, which represents an increase of approximately 300%. However, because of the higher future number of rTSA, which was associated with a lower RB, the overall estimated RB will decline. For better comparison, we also calculated the number of revision procedures using a constant-rate approach. Here, we calculated the highest number of revision procedures of 4100 (95% CI 3568–4852, monotone B-spline approach) and 10,500 (95% CI 8817–12,558, Poisson modeling), respectively, which represents a 2.5 to 8.4-fold increase in revision procedures (Figure 6).

## 4. Discussion 

Despite a rising number of projection studies for hip and knee arthroplasties in recent literature, we still lack empirical results on studies for the upper extremities. Although current growth rates for shoulder arthroplasties are reported to be comparable with, or even higher than the growth rates for total hip and knee procedures [8,10], the demand and impact of shoulder arthroplasty have been underestimated in the past. However, based on evolving scientific evidence regarding good functional outcomes and due to an expanding indication spectrum, shoulder arthroplasty has been one of the main focus in orthopedic surgery in recent years. Therefore, we opted for a data-driven model selection, by minimization of model errors to investigate the trends for primary and revision shoulder arthroplasty procedures from 2010 to 2040. Although both models predicted a slightly different total number due to their distinct intrinsic model assumptions, they showed an important trend during the upcoming decades. Using these models, the number of total shoulder replacements was estimated to grow up to 700%, mainly due to rising adoption of rTSA in fracture-related conditions of the elderly. These growth rates are substantially higher than the current rates of hip and knee arthroplasty [2,7], indicating the immense importance for orthopedic surgeons in the future. 

With the total number and incidence of primary TSA procedures rising, we also modeled a rising number of revision procedures, highlighting its increasing burden in the future.

### 4.1. Projections of Shoulder Arthroplasty and International Comparison

When comparing our projections with other countries, we found that the trend of a rising volume of primary shoulder arthroplasties has also been reported in the United States [10,23,24]. Schwartz et al. detected an over five-fold increase in primary shoulder arthroplasty between 2001 and 2010 [24], while Wagner et al. projected even higher growth rates in upper extremity arthroplasty compared with total hip and knee procedures by the year 2025 (+235% in total volume) [25]. Padegimas et al. even projected the future volume to increase by 755% by 2030. Similar to our results, this can mainly be attributed to an exponential rise in the number of reverse shoulder arthroplasty in the upcoming years, with its prevalence already rising from 7.3 to 19.1 per 100,000 in the U.S. However, the demographic and economic characteristics of the United States cannot easily be transferred to other industrialized countries, although the global demographic pattern of an aging population is identical around the world. However, projections like those of Wagner et al. [25] tend to be overestimated due to a steep increase of procedure volume in the baseline years- a problem we also faced using a Poisson model. For this reason, we implemented an alternative methodical approach, which included a rather asymptotic development of shoulder arthroplasty in the future but still estimated an increase in incidence that is several times higher than the projected increase in hip or knee arthroplasty [2,4,5,26]. 

These findings are also in line with the most national recent trends for shoulder arthroplasty in Europe. Although population and economic data, as well as procedure selection for the major indications, vary between most countries, Lübbeke et al. [13] reported a strong upward trajectory in the incidence of shoulder arthroplasty in nine registries, concluding that due to growing demand, increasing health care capacity, and/or expanding indications, countries could expect to see this continuation in growth in the future. According to the most recent European study of Villate et al. derived from the French hospital discharge database, the number of total shoulder arthroplasties is projected to rise up to 322% by 2050, representing a future challenge for their healthcare system [27]. 

### 4.2. Projections as a Function of Age and Indication

Based on our data projections, this trend is mainly attributed to a considerably increasing utilization and incidence of rTSA in patients older than 80 years, although the number and incidence of procedures performed for younger patients are forecast to rise in the future as well.

While this observed trend is in line with the findings from other countries [13,28], including the United States [9], it is in contrast with the trend seen in hip and knee arthroplasties, where the incidence rate in younger and older patients remained constant over all age groups [2]. This difference may be explained by various factors. First, based on the recent population growth in Germany, the share of patients > 65 years of age is projected to increase from 21% to 30% by the year 2040, which may contribute to a rapid increase in rTSA utilization, as reverse prostheses are rarely implanted in younger patients. Second, the indications for rTSA have been expanding during the last decade, as indicated by the almost ten-fold increase in the number of proximal humerus fractures in this study. Many surgeons may also now see it as a possible solution for poor shoulder conditions, which were previously seen as unsolvable [29]. In complex proximal humerus fractures, which represent the third-most common fracture seen in patients aged > 60 years [30], rTSA was found to provide more reproducible function with better recovery, as well as lower revision rates than HA [31,32]. However, recent studies also suggest, that shoulder arthroplasty due to fracture could be associated with more inconsistent outcomes [33,34] and an increased risk of postoperative complications compared with OA and cuff arthropathy [35]. Additionally, shoulder arthroplasty for fracture seems to lead to an even higher resource utilization [36], which is especially important as we documented a massive increase in these procedures in the upcoming decades, probably affecting future healthcare costs. Against the background of moving toward the era of bundled payment models, an appropriate risk adjustment based on the indication of surgery should, therefore, be promoted to maintain the current standard of care for all patients. 

Although we documented a massive increase in fracture-related rTSA in our study, current scientific evidence contrarily suggests, that non-operative treatment may be favorable in the elderly compared to surgical treatment in certain fracture patterns [37,38]. However, based on the increasing demand for self-independency in the elderly, there is still a controversial debate, on which patients may be better treated by surgical intervention. Therefore, it remains to be seen, if this trend may influence the treatment choice and amount of implantations in the future, as the number of proximal humerus fractures will be constantly rising in many European countries over the next decade [39,40]. 

### 4.3. Projections of Revision Shoulder Arthroplasty

With the total number and incidence of primary TSA procedures rising, we also modeled a rising number of revision procedures, which has globally never been performed up to date. While we were not able to calculate survival or revision rates because of the aforementioned issue, we used the RB as a surrogate parameter to assess the procedure-specific risk for revision. As a consequence, the actual revision risk, especially for TSA, appears to be underestimated by our calculations, which is why we also used a constant rate approach to estimate the number of revision surgeries based on the RB during the baseline years, which still led to an up to 8.4-fold increase by 2040.

Based on our models, revision surgery for TSA will be significant in 2040 and would be expected to increase even more over time as a direct result of the maturation of the increased number of primary surgeries being performed. This raises the concern if there will be enough resources and trained surgeons to carry out these difficult revision procedures in the future. Against the background of a rising number of fracture-related rTSA, this also highlights the enormous need for adequate fracture-prevention programs, in order to limit the number of revision surgeries in the future, as well. 

### 4.4. Study Limitations

The current study has some limitations. First, although the German population forecasts provided rather good agreement between predicted numbers and confirmed past numbers [2,20], they are typically based on hypothetical future assumptions and are, therefore, potentially uncertain. Second, this type of study was based on the procedural growth trajectory for the years 2010–2019, as a longer time frame was not possible due to coding changes after 2009 and the occurrence of the COVID pandemic after 2019, assuming that this trend will continue. However, it is possible, that after the world has overcome all issues regarding the pandemic, new technologies, surgical innovations, or disruptive innovation techniques, such as cartilage regeneration, tissue engineering, or drug therapies that limit the progression of osteoarthritis or enhance fracture recovery or joint-preserving surgery [41]; as well as health-care reforms or reimbursement changes, will meaningfully affect the relationship between supply and demand for shoulder arthroplasty. Especially, it remains to be seen, if the demand of rTSA for proximal humerus fracture will actually see the projected rise in the future, as more and more studies suggest that non-operative treatment may be more favorable in the elderly compared to a surgical approach [37,38,42].

Overall, in contrast to clinical studies, like randomized controlled trials, which draw evidence from (allegedly) artificially generated data, large database studies, like the presented study, describe results of the actual treatment reality. The current study aims to analyze the current practice and its changes over time, giving the reader an unselected view on surgical demand, which cannot be obtained from case series or prospective studies. Therefore, it has to be seen as an important additive or complementary instrument to clinical studies, providing the possibility to examine the potential effects of current scientific evidence on the daily surgical routine on the one side and to analyze treatment trends, whose causal links can be further analyzed by randomized-controlled studies, on the other side.

## 5. Conclusions

The current study demonstrates that using a data-driven modeling approach, the rate of both primary and revision shoulder arthroplasty procedures is projected to rapidly increase during the next 20 years, with the rate of fracture-related rTSA, performed in elderly patients, showing the greatest impact of all procedures. In light of limited resources and healthcare budgets, this emphasizes the need for adequate prevention programs on the one side, but also for qualified surgeons to meet the demand and for future research to improve the reliability and survivorship of shoulder arthroplasties on the other side.

## Figures and Tables

**Table 1:** Two-year forecast accuracy using an out-of-sample training-test validation set (minor numbers indicating greater forecast quality).

Model	MSE	RMSE	MAPE	U1	U2
Poisson	48,816.735	220.945	6.595	0.018	0.009
B-spline	19,724.682	140.445	3.108	0.012	0.006

MSE: mean squared error; RMSE: root mean squared error; MAPE: mean absolute percentage error; U1/2: Thiel’s U inequality coefficients.

## References

[1] Nowossadeck E. (2012). Population aging and hospitalization for chronic disease in Germany. Dtsch. Arztebl. Int..

[2] Pilz V., Hanstein T., Skripitz R. (2018). Projections of primary hip arthroplasty in Germany until 2040. Acta Orthop..

[3] Inacio M.C.S., Graves S.E., Pratt N.L., Roughead E.E., Nemes S. (2017). Increase in Total Joint Arthroplasty Projected from 2014 to 2046 in Australia: A Conservative Local Model with International Implications. Clin. Orthop. Relat. Res..

[4] Nemes S., Gordon M., Rogmark C., Rolfson O. (2014). Projections of total hip replacement in Sweden from 2013 to 2030. Acta Orthop..

[5] Kurtz S.M., Lau E., Ong K., Zhao K., Kelly M., Bozic K.J. (2009). Future young patient demand for primary and revision joint replacement: National projections from 2010 to 2030. Clin. Orthop. Relat. Res..

[6] Sloan M., Premkumar A., Sheth N.P. (2018). Projected Volume of Primary Total Joint Arthroplasty in the U.S., 2014 to 2030. J. Bone Jt. Surg. Am..

[7] Rupp M., Lau E., Kurtz S.M., Alt V. (2020). Projections of Primary TKA and THA in Germany from 2016 through 2040. Clin. Orthop. Relat. Res..

[8] Day J.S., Lau E., Ong K.L., Williams G.R., Ramsey M.L., Kurtz S.M. (2010). Prevalence and projections of total shoulder and elbow arthroplasty in the United States to 2015. J. Shoulder Elbow Surg..

[9] Padegimas E.M., Maltenfort M., Lazarus M.D., Ramsey M.L., Williams G.R., Namdari S. (2015). Future patient demand for shoulder arthroplasty by younger patients: National projections. Clin. Orthop. Relat. Res..

[10] Kim S.H., Wise B.L., Zhang Y., Szabo R.M. (2011). Increasing incidence of shoulder arthroplasty in the United States. J. Bone Jt. Surg. Am..

[11] Coleman D., Rowthorn R. (2011). Who’s afraid of population decline? A critical examination of its consequences. Popul. Dev. Rev..

[12] Bengtsson T., Scott K. (2011). Population aging and the future of the welfare state: The example of Sweden. Popul. Dev. Rev..

[13] Lübbeke A., Rees J.L., Barea C., Combescure C., Carr A.J., Silman A.J. (2017). International variation in shoulder arthroplasty. Acta Orthop..

[14] Klug A., Gramlich Y., Wincheringer D., Schmidt-Horlohé K., Hoffmann R. (2019). Trends in surgical management of proximal humeral fractures in adults: A nationwide study of records in Germany from 2007 to 2016. Arch. Orthop. Trauma Surg..

[15] Luciani P., Farinelli L., Procaccini R., Verducci C., Gigante A. (2019). Primary reverse shoulder arthroplasty for acute proximal humerus fractures: A 5-year long term retrospective study of elderly patients. Injury.

[16] Ferrel J.R., Trinh T.Q., Fischer R.A. (2015). Reverse total shoulder arthroplasty versus hemiarthroplasty for proximal humeral fractures: A systematic review. J. Orthop. Trauma.

[17] Jonsson E.Ö., Ekholm C., Salomonsson B., Demir Y., Olerud P. (2021). Reverse total shoulder arthroplasty provides better shoulder function than hemiarthroplasty for displaced 3- and 4-part proximal humeral fractures in patients aged 70 years or older: A multicenter randomized controlled trial. J. Shoulder Elbow Surg..

[18] Gauci M.-O., Cavalier M., Gonzalez J.-F., Holzer N., Baring T., Walch G., Boileau P. (2020). Revision of failed shoulder arthroplasty: Epidemiology, etiology, and surgical options. J. Shoulder Elbow Surg..

[19] DIMDI (2010–2019). Systematisches Verzeichnis—Operationen-und Prozedurenschlüssel—Internationale Klassifikation der Prozeduren in der Medizin (OPS).

[20] Pötzsch O.R.F. (2015). Demographic Analyses, Methods and Projections, Births and Deaths: Germany’s Population by 2060—Results of the 13th Coordinated Population Projection.

[21] Bliemel F. (1973). Theil’s Forecast Accuracy Coefficient: A Clarification. J. Mark. Res..

[22] Hollatz M.F., Stang A. (2014). Nationwide shoulder arthroplasty rates and revision burden in Germany: Analysis of the national hospitalization data 2005 to 2006. J. Shoulder Elbow Surg..

[23] Harjula J.N.E., Paloneva J., Haapakoski J., Kukkonen J., Äärimaa V. (2018). Increasing incidence of primary shoulder arthroplasty in Finland—A nationwide registry study. BMC Musculoskelet. Disord..

[24] Schwartz B.E., Savin D.D., Youderian A.R., Mossad D., Goldberg B.A. (2015). National trends and perioperative outcomes in primary and revision total shoulder arthroplasty: Trends in total shoulder arthroplasty. Int. Orthop..

[25] Wagner E.R., Farley K.X., Higgins I., Wilson J.M., Daly C.A., Gottschalk M.B. (2020). The incidence of shoulder arthroplasty: Rise and future projections compared with hip and knee arthroplasty. J. Shoulder Elbow Surg..

[26] Nemes S., Rolfson O., W-Dahl A., Garellick G., Sundberg M., Kärrholm J., Robertsson O. (2015). Historical view and future demand for knee arthroplasty in Sweden. Acta Orthop..

[27] Villatte G., Erivan R., Barth J., Bonnevialle N., Descamps S., Boisgard S. (2020). Progression and projection for shoulder surgery in France, 2012-2070: Epidemiologic study with trend and projection analysis. Orthop. Traumatol. Surg. Res..

[28] Bayona C.E.A., Somerson J.S., Matsen F.A. (2018). The utility of international shoulder joint replacement registries and databases: A comparative analytic review of two hundred and sixty one thousand, four hundred and eighty four cases. Int. Orthop..

[29] García-Fernández C., Lopiz Y., Rizo B., Serrano-Mateo L., Alcobía-Díaz B., Rodríguez-González A., Marco F. (2018). Reverse total shoulder arhroplasty for the treatment of failed fixation in proximal humeral fractures. Injury.

[30] Court-Brown C.M., Garg A., McQueen M.M. (2001). The epidemiology of proximal humeral fractures. Acta Orthop. Scand..

[31] Gallinet D., Ohl X., Decroocq L., Dib C., Valenti P., Boileau P. (2018). Is reverse total shoulder arthroplasty more effective than hemiarthroplasty for treating displaced proximal humerus fractures in older adults? A systematic review and meta-analysis. Orthop. Traumatol. Surg. Res..

[32] Noguera L., Trigo L., Melero V., Santana F., Torrens C. (2019). Reverse shoulder arthroplasty for acute proximal humeral fractures: Postoperative complications at 7 days, 90 days and 1 year. Injury.

[33] Lindbloom B.J., Christmas K.N., Downes K., Simon P., McLendon P.B., Hess A.V., Mighell M.A., Frankle M.A. (2019). Is there a relationship between preoperative diagnosis and clinical outcomes in reverse shoulder arthroplasty? An experience in 699 shoulders. J. Shoulder Elbow Surg..

[34] Coscia A.C., Matar R.N., Espinal E.E., Shah N.S., Grawe B.M. (2020). Does Preoperative Diagnosis Impact Patient Outcomes Following Reverse Total Shoulder Arthroplasty? A Systematic Review. J. Shoulder Elbow Surg..

[35] Lung B.E., Kanjiya S., Bisogno M., Komatsu D.E., Wang E.D. (2019). Preoperative indications for total shoulder arthroplasty predict adverse postoperative complications. JSES Open Access.

[36] Malik A.T., Bishop J.Y., Neviaser A.S., Beals C.T., Jain N., Khan S.N. (2019). Shoulder Arthroplasty for a Fracture Is Not the Same as Shoulder Arthroplasty for Osteoarthritis: Implications for a Bundled Payment Model. J. Am. Acad. Orthop. Surg..

[37] Fu B.-S., Jia H.-L., Zhou D.-S., Liu F.-X. (2019). Surgical and Non-Surgical Treatment for 3-Part and 4-Part Fractures of the Proximal Humerus: A Systematic Review of Overlapping Meta-Analyses. Orthop. Surg..

[38] Soler-Peiro M., García-Martínez L., Aguilella L., Perez-Bermejo M. (2020). Conservative treatment of 3-part and 4-part proximal humeral fractures: A systematic review. J. Orthop. Surg. Res..

[39] Hemmann P., Ziegler P., Konrads C., Ellmerer A., Klopfer T., Schreiner A.J., Bahrs C. (2020). Trends in fracture development of the upper extremity in Germany-a population-based description of the past 15 years. J. Orthop. Surg. Res..

[40] Martinez-Huedo M.A., Jiménez-García R., Mora-Zamorano E., Hernández-Barrera V., Villanueva-Martinez M., Lopez-de-Andres A. (2017). Trends in incidence of proximal humerus fractures, surgical procedures and outcomes among elderly hospitalized patients with and without type 2 diabetes in Spain (2001-2013). BMC Musculoskelet. Disord..

[41] Baertl S., Alt V., Rupp M. (2020). Surgical enhancement of fracture healing—Operative vs. nonoperative treatment. Injury.

[42] Gomberawalla M.M., Miller B.S., Coale R.M., Bedi A., Gagnier J.J. (2013). Meta-analysis of joint preservation versus arthroplasty for the treatment of displaced 3- and 4-part fractures of the proximal humerus. Injury.

